# The Role of Chromatid Interference in Determining Meiotic Crossover Patterns

**DOI:** 10.3389/fpls.2021.656691

**Published:** 2021-03-09

**Authors:** Marie Sarens, Gregory P. Copenhaver, Nico De Storme

**Affiliations:** ^1^Laboratory for Plant Genetics and Crop Improvement, Division of Crop Biotechnics, Department of Biosystems, Katholieke Universiteit Leuven, Leuven, Belgium; ^2^Department of Biology and the Integrative Program for Biological and Genome Sciences, University of North Carolina at Chapel Hill, Chapel Hill, NC, United States

**Keywords:** chromatid interference, meiotic recombination, crossovers, *Arabidopsis thaliana*, genetic variation

## Abstract

Plants, like all sexually reproducing organisms, create genetic variability by reshuffling parental alleles during meiosis. Patterns of genetic variation in the resulting gametes are determined by the independent assortment of chromosomes in meiosis I and by the number and positioning of crossover (CO) events during meiotic recombination. On the chromosome level, spatial distribution of CO events is biased by multiple regulatory mechanisms, such as CO assurance, interference and homeostasis. However, little is known about how multiple COs are distributed among the four chromatids of a bivalent. Chromatid interference (CI) has been proposed as a regulatory mechanism that biases distribution of multiple COs toward specific chromatid partners, however, its existence has not been well-studied and its putative mechanistic basis remains undescribed. Here, we introduce a novel method to quantitatively express CI, and take advantage of available tetrad-based genotyping data from Arabidopsis and maize male meiosis to quantify CI effects on a genome-wide and chromosomal scale. Overall, our analyses reveal random involvement of sister chromatids in double CO events across paired chromosomes, indicating an absence of CI. However, on a genome-wide level, CI was found to vary with physical distance between COs, albeit with different effects in Arabidopsis and maize. While effects of CI are minor in Arabidopsis and maize, the novel methodology introduced here enables quantitative interpretation of CI both on a local and genome-wide scale, and thus provides a key tool to study CI with relevance for both plant genetics and crop breeding.

## Introduction

Meiosis is a specialized cell division that reduces ploidy by half and generates cells essential for sexual reproduction. It consists of a single round of pre-meiotic DNA replication, followed by two consecutive rounds of chromosome segregation, in which homologous chromosomes separate in meiosis I, and sister chromatids separate in meiosis II, to yield four daughter cells. Together with this ploidy reduction, meiosis also creates novel allelic combinations by reshuffling parental DNA through independent assortment and homologous recombination. Meiotic recombination occurs during prophase I and involves pairing and synapsis of homologous chromosomes, followed by the reciprocal exchange of genetic information via crossovers (COs), which are cytologically manifested as chiasmata (Janssens, 1909; Hunter, 2015).

Despite its complexity, key elements of meiotic recombination are conserved across eukaryotes. It is initiated by the programmed induction of double-strand breaks (DSBs) catalyzed by the conserved topoisomerase-like protein SPO11 together with several other associated proteins (Keeney et al., 1997; De Muyt et al., 2007, 2009; Vrielynck et al., 2016). Subsequent processing of DSBs by the MRN/X complex results in the formation of 3′ single-stranded DNA ends, which recognize and invade the homologous chromosome to enable DNA repair via the non-sister chromatid. This single-end invasion (SEI) is facilitated by the meiosis-specific recombinase DMC1 and RAD51, and results in the formation of a D-loop intermediate (Da Ines et al., 2013; Brown and Bishop, 2015). These SEI intermediates are unstable and frequently dissociate to be repaired by the synthesis-dependent strand annealing (SDSA) pathway to yield non-crossovers (McMahill et al., 2007). Some D-loops persist and are further processed by annealing to the 3′ single-stranded DNA end on the other side of the original break in a process called second-end capture. The resulting intermediate structure physically interlinks both homologs and after ligation of adjacent DNA ends goes on to form a double Holliday Junction (dHJ). Finally, endonucleases called resolvases cleave the dHJs and the primary product of this type of resolution are CO events, as evidenced in *Saccharomyces cerevisiae* (Allers and Lichten, 2001; Mercier et al., 2015; Wang and Copenhaver, 2018).

**Figure 1:**
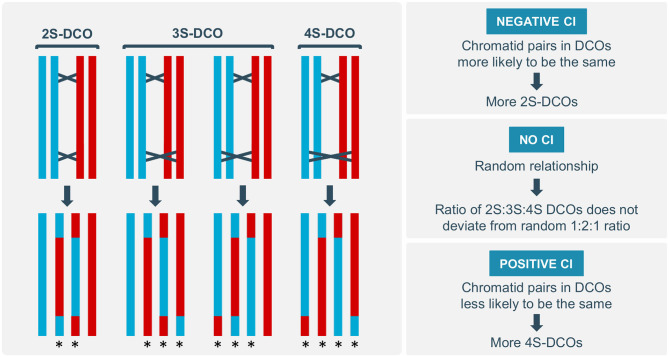
Different types of double crossovers (DCOs) and chromatid interference (CI). DCOs can occur between the same pair of chromatids (two-strand or 2S-DCO), resulting in two recombinant and two parental chromosomes. DCOs can have only one chromatid in common (three-strand or 3S-DCO), resulting in three recombinant and one parental chromosome. DCOs can occur between different chromatids (four-strand or 4S-DCO), resulting in four recombinant chromosomes. If the 2S:3S:4S DCO ratio does not significantly deviate from the random 1:2:1 ratio, CI is absent. If there are relatively more 2S-DCOs, negative CI occurs; if there are relatively more 4S-DCOs, positive CI occurs. Recombinant chromosomes are indicated by an asterisk.

Most eukaryotes have an abundance of meiotic DSBs, but relatively few COs. Moreover, COs are non-uniformly distributed across the genome, with many organisms showing a preferential skewing of COs toward the (sub-)telomeric regions (Giraut et al., 2011; Li et al., 2015). Several regulatory phenomena have been found to influence CO distribution on the local, chromosomal and genome-wide scale, including CO assurance, homeostasis and interference (Berchowitz and Copenhaver, 2010; Wang et al., 2015; Wang and Copenhaver, 2018). In contrast, little is known about the regulation of CO distribution among the four chromatids of a paired chromosome set. Chromatid interference (CI) has been proposed as a mechanism that biases distribution of multiple COs within a meiotic bivalent toward specific chromatid partners (Zhao et al., 1995). When two COs occur on the same pair of homologous chromosomes, three different outcomes are possible (Figure 1): (A) both COs use the same non-sister chromatids, resulting in a two-strand double CO (2S-DCO); (B) COs share only one chromatid, resulting in a three-strand double CO (3S-DCO), as can be achieved in two different ways; or (C) COs use a different pair of non-sister chromatids, resulting in a four-strand double CO (4S-DCO). Typically, putative sister chromatid interactions are ignored because they are largely suppressed during meiosis (Schwacha and Kleckner, 1994). Depending on the type of DCOs that is favored in meiosis, different types of CI effects can be defined. In the absence of CI, chromatid partner participation in DCO events occurs randomly, resulting in a 1:2:1 ratio of 2S-, 3S-, and 4S-DCOs. Positive CI occurs when a CO between a pair of chromatids suppresses the occurrence of a second CO between the same two chromatids, resulting in a higher frequency of 4S-DCOs. In contrast, negative CI occurs when the same pair of chromatids is more likely to experience DCOs than what would be expected by a random distribution, resulting in an increased number of 2S-DCOs (Zhao et al., 1995).

The occurrence of CI would influence meiotic CO patterning and the genetic make-up of gametes, and would therefore have a major impact on genetic analyses such as linkage-based mapping and QTL assays. The construction of genetic maps and identification of QTLs relies on measuring the genetic distance between loci in mapping populations (e.g., F2, RIL, NIL, etc.) derived from crossing polymorphic parents. Genetic distance is determined by quantifying the frequency of recombinant genotypes between loci, and is expressed in centiMorgans (cM). Loci on different chromosomes segregate randomly in MI and yield equal fractions of recombinant and non-recombinant genotypes, resulting in a genetic distance of 50 cM. For loci on the same chromosome, however, recombinant genotypes can only result from intervening COs. Genetic map distances are therefore often simple calculations of CO frequency, although more nuanced formulas are available that account for the occurrence of DCOs, such as the Perkins (1949), Haldane (1919), and Kosambi (1944) functions. However, all these mapping functions assume a complete absence of CI and thus integrate a random 1:2:1 ratio of 2S:3S:4S DCOs as a core part of their derivation. This assumption, however, may lead to inaccuracies in the calculation of actual genetic distances. For example, negative CI will create relatively more 2S-DCOs and thus lower the odds of detecting double COs, particularly when loci are located far apart. As a result, negative CI can cause an underestimation of the actual genetic distance, even when calculated using approaches that account for DCOs. By comparison, positive CI results in relatively more 4S-DCOs and increases the odds of detecting DCOs, which can lead to an overestimation of the actual genetic distance. To investigate the significance of these biases and facilitate the development of mapping functions that incorporate the influence of CI, a quantitative measure of CI is needed.

Accurate quantification of CI requires genotypic information from all four meiotic products, referred to as “tetrad analysis.” Many fungi retain their meiotic products in an ascus making tetrad data easy to obtain. Tetrad data is harder to gather from most multicellular organisms because their meiotic products do not remain attached in their meiotic configuration. Despite these difficulties, tetrad analysis has been achieved in several multicellular species, including plants, allowing analysis of CI. In Arabidopsis, mutations in the *QRT1* gene cause mature pollen grains to remain together in their original meiotic tetrad configuration (Preuss et al., 1994). Backcrossing single pollen tetrads from *qrt1* F1 plants from crosses between two polymorphic parents generates four progeny plants that can be analyzed to determine the genotype of the original tetrad (Copenhaver et al., 1998; Lu et al., 2012; Wijnker et al., 2013; Liu et al., 2018). Several plant species produce natural tetrads at the mature pollen stage (Copenhaver, 2005), but even in those that do not tetrad data can be obtained by isolating and genotyping individual microspores from single tetrads at the end of sporogenesis. The latter approach has been used to obtain high resolution CO data from maize male meioses (Li et al., 2015). In human oocytes, tetrad data has been obtained by isolating and sequencing the first and second polar bodies and the female pronucleus (Hou et al., 2013). In mouse, tetrad analysis has been performed for both male and female meiosis. In female mice this is achieved by microdissection of individual germinal vesicle-stage oocytes from F1 hybrids. In male mice, tetrad data was obtained by isolating late prophase I primary spermatocytes using flow cytometry (Cole et al., 2014).

CI has been examined in *Saccharomyces cerevisiae, Neurospora crassa, Aspergillus nidulans, Arabidopsis thaliana, Zea mays*, and humans (Lindegren and Lindegren, 1942; Strickland, 1957; Hawthorne and Mortimer, 1960; Zhao et al., 1995; Copenhaver et al., 1998; Chen et al., 2008; Hou et al., 2013; Li et al., 2015). These studies suggest weak or no CI with minor variation across eukaryotic phyla. In contrast, a recent study using *in situ* cytological probing of meiotic chromosomes provided direct evidence for strong positive CI (i.e., 64% 4S-DCOs instead of the expected 25%) in two interspecific hybrids, *Lolium multiflorum* × *Festuca pratensis* and *Allium cepa* × *A. roylei* (Ferreira et al., 2020). These findings are striking as they indicate that strong CI may occur in plant meiosis, and that this may be influenced by genetic background, and particularly hybrid status. Some studies have also examined the influence of the centromere on CI. In humans, weak negative CI is present within chromosome arms, but no CI effects are detected when the two COs are on the opposite sides of a centromere (Hou et al., 2013). In contrast, in maize, weak CI was observed for DCOs within one chromosome arm as well as for DCOs spanning the centromere (Li et al., 2015).

Prior CI analyses are limited to accepting or rejecting a fit to the expected 1:2:1 ratio and are mainly focused on genome-wide effects, leaving many questions unanswered, including: “If CI is present, does it show variation in strength across chromosomes?,” “Is CI subject to chromosome-specific or location-specific effects?,” “Does inter-CO distance influence CI and is there a link with CO interference?,” and “Does CI differ between male and female meiosis?.” In this study, we introduce a novel analytical framework for the quantitative interpretation of CI and apply this to tetrad-based genotyping data from Arabidopsis and maize male meiosis to unravel putative, yet unexplored CI effects.

## Analysis of Chromatid Interference in Arabidopsis and Maize Male Meiosis

We quantified CI in Arabidopsis using previously acquired tetrad-based genotyping data of male meiosis (Copenhaver et al., 1998). This dataset contains PCR-based marker genotypes from 58 meiotic tetrads for chromosome 1, 3, and 5 and from 143 tetrads for chromosome 2 and 4 (see Supplementary Data Sheet 1). Additional information on the number of markers used can be found in Supplementary Table 1. We measured CI in maize using tetrad-based genotyping data of male meioses provided by Li et al. (2015). This dataset consists of single-microspore sequencing data of 24 tetrads and provides high-resolution genotyping data of corresponding meiotic events with an average of 271,524 SNPs per tetrad. The corresponding Arabidopsis and maize datasets were used to identify all DCOs and to determine levels of CI. For Arabidopsis, the dataset contains a total of 843 COs, including 385 DCOs (Table 1). For maize, the dataset contains a total of 924 COs, and 684 DCOs (Table 2).

**Table 1:** Analysis of chromatid interference (CI) in Arabidopsis male meiosis.

	**Whole chromosome**			**Same arm**			**Different arm**		
	**Total DCOs**	**Observed2S:3S:4S ratio(Expected ratio)**	**CI value**	**Total DCOs**	**Observed 2S:3S:4S ratio (Expected ratio)**	**CIvalue**	**Total DCOs**	**Observed 2S:3S:4S ratio(Expected ratio)**	**CI value**
Chr1	101	33:44:24(25.25:50.5:25.25)	−0.09	49	18:20:11 (12.25:24.5:12.25)	−0.14	52	15:24:13(13:26:13)	−0.04
Chr2	98	24:52:22(24.5:49:24.5)	−0.02	53	12:23:18 (13.25:26.5:13.25)	0.11	45	12:29:4 (*)(11.25:22.5:11.25)	−0.18 (*)
Chr3	66	20:33:13(16.5:33:16.5)	−0.11	23	9:9:5 (5.75:11.5:5.75)	−0.17	43	11:24:8(10.75:21.5:10.75)	−0.07
Chr4	69	20:28:21(17.25:34.5:17.25)	0.01	30	12:9:9 (7.5:15:7.5)	−0.10	39	8:19:12(9.75:19.5:9.75)	0.10
Chr5	51	15:21:15(12.75:25.5:12.75)	0	14	4:5:5 (3.5:7:3.5)	0.07	37	11:16:10(9.25:18.5:9.25)	−0.03
Total	385	112:178:95(96.25:192.5:96.25)	−0.04	169	55:66:48 (*) (42.25:84.5:42.25)	−0.04	216	57:112:47(54:108:54)	−0.05

*CI is determined using both the 2S:3S:4S DCO ratio method and the novel quantitative analysis method (CI value). Results are shown for DCOs along the chromosomes, as well as for single-arm DCOs and for DCOs spanning a centromere. In the absence of CI, a random 2S:3S:4S DCO ratio of 1:2:1 and a CI value of 0 is expected. Deviations from the random 1:2:1 ratio were statistically analyzed using the Chi-Square test of goodness-of-fit (when total amount of DCOs ≥ 20) or using the exact multinomial test (when total amount of DCOs <20). Deviations of the CI value were statistically tested using the Wilcoxon signed rank test. P-values of all statistical tests are provided in Supplementary Table 2. Statistical tests were corrected via multiple penalty testing using Bonferroni correction (α = 0.008). Significant results before correcting are indicated with an asterisk. Results are based on PCR-based tetrad genotyping data (Copenhaver et al., 1998)*.

**Table 2:** Analysis of chromatid interference (CI) in maize male meiosis.

	**Whole chromosome**			**Same arm**			**Different arm**		
	**Total DCOs**	**Observed2S:3S:4S ratio(Expected ratio)**	**CI value**	**Total DCOs**	**Observed 2S:3S:4S ratio (Expected ratio)**	**CIvalue**	**Total DCOs**	**Observed2S:3S:4S ratio(Expected ratio)**	**CI value**
Chr1	110	23:57:30(27.5:55:27.5)	0.06	86	18:47:21 (21.5:43:21.5)	0.04	24	5:10:9(6:12:6)	0.17
Chr2	83	17:47:19(20.75:41.5:20.75)	0.02	59	10:31:18 (14.75:29.5:14.75)	0.14	24	7:16:1(6:12:6)	−0.25 (*)
Chr3	78	15:52:11 (*)(19.5:39:19.5)	−0.05	54	8:36:10 (*) (13.5:27:13.5)	0.04	24	7:16:1(6:12:6)	−0.25 (*)
Chr4	71	15:39:17(17.75:35.5:17.75)	0.03	48	6:29:13 (12:24:12)	0.15	23	9:10:4(5.75:11.5:5.75)	−0.22
Chr5	77	18:44:15(19.25:38.5:19.25)	−0.04	53	12:34:7 (13.25:26.5:13.25)	−0.09	24	6:10:8(6:12:6)	0.08
Chr6	64	23:25:16(16:32:16)	−0.11	53	21:21:11 (*) (13.25:26.5:13.25)	−0.19 (*)	11	2:4:5(2.75:5.5:2.75)	0.27
Chr7	56	14:25:17(14:28:14)	0.05	32	8:13:11 (8:16:8)	0.09	24	6:12:6(6:12:6)	0
Chr8	61	11:34:16(15.25:30.5:15.25)	0.08	37	8:19:10 (9.25:18.5:9.25)	0.05	24	3:15:6(6:12:6)	0.13
Chr9	43	12:21:10(10.75:21.5:10.75)	−0.05	20	5:11:4 (5:10:5)	−0.05	23	7:10:6(5.75:11.5:5.75)	−0.04
Chr10	41	12:22:7(10.25:20.5:10.25)	−0.12	18	6:8:4 (4.5:9:4.5)	−0.11	23	6:14:3(5.75:11.5:5.75)	−0.13
Total	684	160:366:158(171:342:171)	−0.003	460	102:249:109 (101.5:203:101.5)	0.02	224	58:117:49(56:112:56)	−0.04

*Presence of CI is determined using both the 2S:3S:4S DCO ratio method and the novel quantitative analysis method (CI value). Results are shown for DCOs along the chromosomes, as well as for same-arm DCOs and DCOs that span a centromere. In case CI is absent, a DCO ratio of 1:2:1 and a CI value of 0 is expected. Deviations from the expected 1:2:1 ratio were statistically tested using a Chi-Square test of goodness-of-fit (when total number of DCOs ≥ 20) and via an exact multinomial test (when total number of DCOs <20). Deviations of the CI value were statistically tested using the Wilcoxon signed rank test. P-values of all statistical tests are provided in Supplementary Table 6. Statistical tests were corrected via multiple penalty testing using Bonferroni correction (α = 0.0045). Significant results before correcting are indicated with an asterisk. Results are based on genotyping data from Li et al. (2015)*.

### Novel Approach for Quantifying CI

Traditionally, CI is assessed by testing goodness-of-fit to the expected 1:2:1 ratio of 2S:3S:4S DCOs for individual genomic intervals (Figure 1). This method enables hypothesis testing for the presence or absence of CI but does not allow interpretation of the strength of CI when it is detected. Without a quantitative value for CI, it is difficult to compare different genomic intervals when trying to ascertain local variation, to interpret the impact of genetic or physical distance on CI, or to assess the putative effect of structural domains, such as the centromere. Additionally, the goodness-of-fit method does not allow easily interpretable graphical representations of putative CI dynamics and thus limits studies on the occurrence, relevance and regulation of CI.

To address this, we have developed a novel approach for CI analysis that relies on a quantitative parameter calculated from the number of DCO types in a specific genomic interval, and thus enables interpretation of both the strength and direction of CI in a local, chromosomal or genome-wide context. This is achieved by assigning discrete values to each DCO type within an interval and by calculating the mean CI value of all DCOs. The specific CI value for each DCO type is determined according to the following principle. If a chromatid participating in a first CO does not participate in the second CO of the DCO, it is assigned a value of 0.5. In the opposite case, if a chromatid participating in a first CO also participates in the second CO, it is assigned a value of −0.5. The CI value of a specific DCO type is hence obtained by the summed values assigned to the two non-sister chromatids involved in the first CO. Using this approach, 2S-DCOs are assigned a value of −1, 3S-DCOs are assigned a value of 0 and 4S-DCOs are assigned a value of 1. Averaging the assigned values of all DCOs within a specific genomic interval gives a mean CI value between −1 and 1, providing a quantitative measure for the strength and direction of CI. A mean CI value of −1 indicates complete negative CI (i.e., all DCOs are 2S type), a value of 1 indicates complete positive CI (i.e., all DCOs are 4S type), and a CI value of 0 indicates absence of CI.

### Genome-Wide and Chromosome-Specific Levels of CI

We examined the presence and direction of CI for the complete genome and each chromosome using both the traditional ratio-based analysis and our novel quantitative analysis method (indicated as “CI value”). For Arabidopsis, the ratio of 2S:3S:4S DCOs for all five chromosomes does not deviate significantly from the random 1:2:1 distribution (Table 1 and Supplementary Table 2), indicating absence of CI on a genome-wide and chromosome-specific scale, as was previously reported (Copenhaver et al., 1998). Consistent with this, quantitative determination of CI across the genome and for all individual chromosomes yields CI values that do not significantly differ from 0 (Table 1 and Supplementary Table 2). These findings demonstrate that Arabidopsis male meiosis does not experience any bias toward a specific DCO type on a genome-wide or chromosome-specific level.

We also tested the hypothesis that the centromere influences CI. For this, DCOs were split into two groups: DCOs that occur on the same chromosome arm, and DCOs that span the centromere. On the genome-wide level, we detected a significant deviation from the 1:2:1 ratio for all DCOs occurring on the same chromosome arm (55:66:48, Chi-Square test of goodness-of-fit, *P* = 0.013). However, the corresponding CI value did not differ significantly from 0, indicating that this deviation is neutral (Table 1 and Supplementary Table 2). For DCOs spanning the centromere, we observed a significant bias for chromosome 2 for both the ratio (12:29:4, Chi-Square test of goodness-of-fit, *P* = 0.037) and the CI value (−0.18, Wilcoxon signed rank test, *P* = 0.024), indicating presence of negative CI. However, after applying a multiple testing penalty using Bonferroni correction these deviations were no longer significant. All these results are based on PCR-based genotyping data, and it is possible that DCOs were undercounted, for example by missing terminally located COs for which no flanking markers were available. To address this, we examined two additional Arabidopsis datasets (Wijnker et al., 2013; Liu et al., 2018) that contain tetrad-based sequencing data from 22 male meiotic tetrads. Aalysis of CI using only these datasets is not informative because the number of DCOs is too low (Supplementary Tables 3, 4). However, merging these datasets with the marker-based genotyping dataset (Copenhaver et al., 1998) did not change prior results, with absence of significant deviations for all chromosomes (Supplementary Table 5), indicating that the marker-based data enables reliable interpretation of CI.

In maize, the genome-wide ratio of 2S:3S:4S DCOs does not deviate significantly from the null hypothesis of a 1:2:1 ratio (Table 2 and Supplementary Table 6), indicating absence of CI. In line with this, quantitative analysis of CI yields a value that does not significantly differ from 0, confirming the results of the ratio-based method. Chromosome-specific analysis of the 2S:3S:4S DCO ratio shows a significant deviation from the random 1:2:1 ratio for chromosome 3 (15:52:11, Chi-Square test of goodness-of-fit, *P* = 0.011), but the corresponding CI value does not differ significantly from 0, indicating that this deviation is neutral and that CI is absent.

Genome-wide, DCOs on the same arm or spanning a centromere in maize do not show CI (Table 2 and Supplementary Table 6). Chromosome-specific ratio-based analyses of CI for same-arm DCOs yield significant deviations from the 1:2:1 ratio for chromosome 3 and 6 (8:36:10 and 21:21:11, respectively, Chi-Square test of goodness-of-fit, *P* = 0.046 and *P* = 0.048, respectively), but for chromosome 3 no significant difference is observed for the CI value. Conversely, for same-arm DCOs on chromosome 6 a negative CI value (−0.19, Wilcoxon signed rank test, *P* = 0.039) is observed. For DCOs that span a centromere, we observe no significant deviation from the 1:2:1 ratio. For the CI value, significant deviations are observed for chromosome 2 and 3 (−0.25 for both, Wilcoxon signed rank test, *P* = 0.021), indicating negative CI. However, after applying a multiple testing penalty (Bonferroni correction) significant differences were no longer observed.

### Effect of Inter-CO Distance on Chromatid Interference

**Figure 2:**
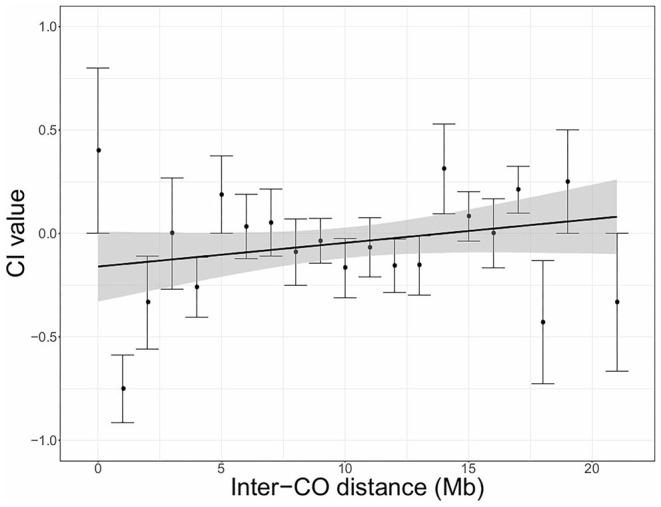
Chromatid interference (CI) in function of physical distance (Mb) between adjacent COs (inter-CO distance) in Arabidopsis male meiosis. Linear regression is performed with the total number of DCOs per inter-CO distance as weighted factors. Gray shaded areas indicate the 95% confidence interval for the regression line. Intercept = −0.1598; Slope = 0.0115; *R*^2^ = 0.0058. Results are based on PCR-based tetrad genotyping data (Copenhaver et al., 1998).

**Figure 3:**
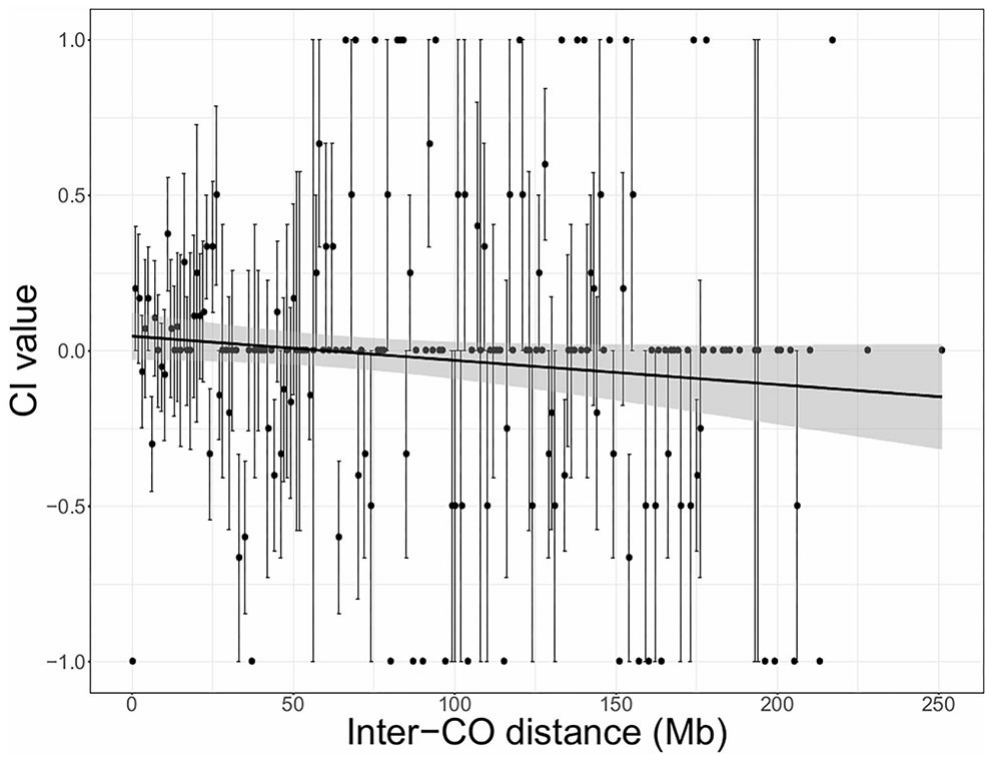
Chromatid interference (CI) in function of physical distance (Mb) between adjacent COs (inter-CO distance) in maize male meiosis. Linear regression is performed with the total number of DCOs per inter-CO distance as weighted factors. Gray shaded areas indicate the 95% confidence interval for the regression line. Intercept = 0.04733; Slope = −0.00078; *R*^2^ = 0.0046. Results are based on sequencing-based tetrad genotyping data (Li et al., 2015).

To test the hypothesis that chromatid interference might vary depending on the distance between two adjacent COs we calculated the level of CI as a function of physical inter-CO distance (Mb) using the new CI quantification method (Figures 2, 3). As marker-based genotyping data does not provide the precise location of recombination events, CO positions were estimated by averaging the genomic location of adjacent recombinant markers. To calculate the inter-CO distance of a DCO, the physical distance between the two COs was then determined and rounded to the closest Mb integer.

For Arabidopsis male meiosis, the genome-wide analysis indicates that CI increases as a function of inter-CO distance (Figure 2). As the presented averaged values are based on different numbers of DCOs, a weighted linear regression was performed. Although correlation is rather weak (*R*^2^ = 0.0058), this analysis reveals a minor influence of inter-CO distance on CI, with negative CI values when inter-CO distances are <14 Mb and positive CI for inter-CO distances of more than 14 Mb. However, as the 95% confidence interval is rather broad and covers the zero baseline, the observed correlation is non-significant. A similar analysis was performed with the merged dataset containing both PCR-based and sequencing-based genotyping data (Supplementary Figure 1). This extended analysis revealed even lower correlation between CI and physical inter-CO distance with the mean CI value for all DCO distances amounting close to 0, further indicating that CI effects do not vary with inter-CO distance.

For maize, genome-wide results indicate positive CI when inter-CO distances are <60 Mb and negative CI when inter-CO distances are larger (Figure 3). However, considering the low correlation value (*R*^2^ = 0.0046), observed effects are non-significant and more high-throughput genotyping data is required to validate these genome-wide trends and to assess for putative chromosome-specific variation.

## Discussion

Chromatid interference (CI), the mechanism that describes the bias of chromatid partner choice in multiple COs, is a poorly studied aspect of meiotic CO patterning. Generally, it is assumed that CI does not exist and that the choice of the specific sister chromatid involved in a CO event occurs randomly and independently of neighboring COs, leading to a balanced 1:2:1 ratio of 2S:3S:4S DCOs (Zhao et al., 1995; Teuscher et al., 2000). However, some studies have reported a bias of this 1:2:1 ratio, indicating the presence of CI, although often rather weak (Zhao et al., 1995; Hou et al., 2013; Li et al., 2015; Ferreira et al., 2020). As presence of CI has consequences for genetic mapping studies and specific breeding applications, more detailed studies are needed to unravel the actual occurrence and relevance of this rather elusive process. In order to facilitate this, we here introduce a novel approach for quantifying and representing CI, and demonstrate its applicability by reassessing the role of CI in meiotic CO patterning in plants by using available tetrad genotyping data from Arabidopsis and maize.

CI is traditionally assessed by testing whether the ratio of 2S:3S:4S DCOs deviates from the expected 1:2:1 ratio (Copenhaver et al., 1998; Hou et al., 2013; Li et al., 2015; Liu et al., 2018; Ferreira et al., 2020). However, this method only verifies the presence or absence of CI, and does not provide straightforward information about its strength or direction. We here extend this basic analysis by introducing a new approach to quantitatively measure CI, enabling the assessment of both the strength and direction of CI. This new approach provides a single quantitative measure of CI for each genomic region, ranging from short intervals to whole chromosomes, and therefore strongly facilitates data interpretation and comparative analysis of CI. This new methodology could be useful in a broad range of studies that are focused on CO patterning, such as those aimed at describing the meiotic CO landscape and resulting genetic variation, as well as those aimed at elucidating the genetic basis and molecular mechanism(s) underlying CI.

Using both the ratio-based method and the CI value, we re-analyzed available tetrad-based genotyping data of Arabidopsis (Copenhaver et al., 1998; Wijnker et al., 2013; Liu et al., 2018) and maize (Li et al., 2015). Our results provide no evidence for genome-wide or chromosome-specific effects of CI in both species. Similarly, no common significant deviations were observed when assessing the effect of the centromere, indicating that the physical peculiarities of the centromere do not restrict or impose biases toward chromatid partner choice in DCO events. For maize, using the same data, Li et al. (2015) reported significant CI for DCOs occurring on the same chromosome arm and DCOs spanning the centromere. However, these conclusions were based on boot-strapping analysis which is different from the Chi-Square test of goodness-of-fit used in this analysis. Strikingly, for both Arabidopsis chromosome 2 and maize chromosomes 2 and 3 a tendency toward negative CI has been observed for DCOs that span the centromere (CI values of −0.20 and −0.25, respectively). These deviations indicate for the potential occurrence of chromosome-specific signatures, either structural or regulatory, that impose a directed CI effect on COs that occur on a different side of the centromere. However, whether these observed CO biases reflect actual CI effects, and if so, by which molecular mechanism(s) this is imposed remains to be further investigated. Overall, our findings are in line with most previous studies, reporting no or only weak presence of CI in different species (Zhao et al., 1995; Copenhaver et al., 1998; Chen et al., 2008; Hou et al., 2013; Li et al., 2015). Interestingly, a recent study reported presence of strong positive CI in two interspecific plant hybrids, *Lolium multiflorum* × *Festuca pratensis* and *Allium cepa* × *A. roylei* (Ferreira et al., 2020). This is the first study that demonstrates a clear bias in the specific configuration of DCOs, and thus provides strong and unambiguous evidence that CI may occur in plant meiosis. However, it is important to note that CI in these grass and Allium species was observed in hybrid genotypes that result from interspecific or -genic hybridization, and thus relates to COs that occur between homeologous chromosomes with putative impact of genomic heterozygosity, structural chromosome variation or differential DNA compaction. As a consequence, it is not clear yet whether these findings can be extrapolated to non-hybrid or intraspecific hybrid genotypes that exhibit regular homology and sufficient compatibility between both parental genomes.

Using the new CI quantification methodology, we also assessed whether CI depends on the physical distance between COs. Due to the general absence of CI on a chromosome-wide level, this question has remained unaddressed in previous studies. However, it may still be possible that there is a bias toward specific sister chromatids when COs occur in close proximity to each other, whereas this may be antagonized by DCOs in which COs are distantly positioned from each other. For both Arabidopsis and maize, we observed variation in CI depending on physical inter-CO distance. For Arabidopsis, there was a tendency toward negative CI when participating COs are closely located to each other and toward positive CI when inter-CO distance becomes larger. In maize, we observed the opposite trend. However, as observed effects were only minor and lacked significant correlation, further investigation using more extended datasets is needed to validate the putative effect of inter-CO distance on CI.

Apart from being influenced by physical inter-CO distance, CI may also exhibit regional/local variation due to specific determinants that act *in cis* (e.g., chromatin status, sister chromatid cohesion, etc.). Such local effects have not yet been analyzed in any species due to lack of dedicated methodology, sufficient data and/or saturated genotyping data. By using our new methodology and applying it for the analysis of large datasets of highly saturated genotyping profiles, local effects of CI on CO patterning may be uncovered and characterized. However, similar as for the traditional ratio-based method, our approach still relies on tetrad-based genotyping data, as information on a large number of DCOs is required to perform reliable data interpretation, and this is in spite of several scientific advances often laborious and time consuming, or in some species even impossible to obtain. Therefore, it remains challenging to study local variation of CI effects, as well other aspects of CI, such as sex-specific differences (i.e., male vs. female meiosis) and temporal dynamics during plant aging and development.

## Data Availability Statement

Publicly available datasets were analyzed in this study. This data can be found here: data from Li et al. (2015) is available at https://www.nature.com/articles/ncomms7648#Sec21; data from Wijnker et al. (2013) is available at https://elifesciences.org/articles/01426/figures#files; data from Liu et al. (2018) is available at https://gattaca.nju.edu.cn/pub_data.html; data from Copenhaver et al. (1998) is provided as Supplementary Material.

## Author Contributions

NDS, GC, and MS contributed to the main conceptual ideas and manuscript outline. MS performed the data analysis and wrote the manuscript with inputs, corrections, and critical feedback from the other authors. All authors contributed to the article and approved the submitted version.

## Conflict of Interest

The authors declare that the research was conducted in the absence of any commercial or financial relationships that could be construed as a potential conflict of interest.
