# Supplementary material for: The Role of Chromatid Interference in Determining Meiotic Crossover Patterns
Source: Front Plant Sci. 2021 Mar 9;12:656691. doi: 10.3389/fpls.2021.656691 (PMC7985435; doi:10.3389/fpls.2021.656691)
Supplement: Supplementary file 1 [file Table_1.DOCX]

**Supplementary Table S1. Information on markers used to perform tetrad-based genotyping in Arabidopsis.** The first column represents the total number of markers that were used per chromosome. For chromosome 2 and 4, all tetrads were genotyped using all markers present. For chromosome 1, 3 and 5, not all tetrads were genotyped using all available markers. The second column represents the average number of markers that were used per tetrad per chromosome (± standard deviation (SD)).

|  | **Total number of markers** | **Average number of markers per tetrad (± SD)** |
| --- | --- | --- |
| **Chr1** | 27 | 17 (± 1.77) |
| **Chr2** | 17 | 17 (± 0) |
| **Chr3** | 20 | 11 (± 2.9) |
| **Chr4** | 21 | 21 (± 0) |
| **Chr5** | 22 | 12 (± 2.4) |
