# Supplementary material for: The Role of Chromatid Interference in Determining Meiotic Crossover Patterns
Source: Front Plant Sci. 2021 Mar 9;12:656691. doi: 10.3389/fpls.2021.656691 (PMC7985435; doi:10.3389/fpls.2021.656691)
Supplement: Supplementary file 2 [file Table_2.DOCX]

**Supplementary Table S2. P-values for analysis of chromatid interference (CI) in Arabidopsis male meiosis.** P-values are shown for both the 2S:3S:4S DCO ratio (Chi-Square test of goodness-of-fit when total number of DCOs ≥ 20 and exact multinomial test when total number of DCOs < 20) and the CI value (Wilcoxon signed rank test), for all DCOs along the chromosomes, as well as for single-arm DCOs and for DCOs spanning a centromere. P-values correspond to data represented in Table 1. Results are based on PCR-based tetrad genotyping data (Copenhaver et al., 1998).

|  | **WHOLE CHROMOSOME** | | **SAME ARM** | | **DIFFERENT ARM** | |
| --- | --- | --- | --- | --- | --- | --- |
|  | **p-value**  **2S:3S:4S ratio** | **p-value**  **CI value** | **p-value**  **2S:3S:4S ratio** | **p-value**  **CI value** | **p-value**  **2S:3S:4S ratio** | **p-value**  **CI value** |
| **Chr1** | 0.1942 | 0.1175 | 0.161 | 0.09896 | 0.7939 | 0.3575 |
| **Chr2** | 0.7989 | 0.3864 | 0.3193 | 0.1393 | 0.03688 | 0.02439 |
| **Chr3** | 0.476 | 0.1135 | 0.29896 | 0.1507 | 0.6065 | 0.2529 |
| **Chr4** | 0.2896 | 0.4409 | 0.06721 | 0.2628 | 0.655 | 0.1913 |
| **Chr5** | 0.452 | 1 | 0.4668 | 0.3949 | 0.6943 | 0.4214 |
| **Total** | 0.1584 | 0.1188 | 0.01303 | 0.2458 | 0.5427 | 0.1639 |
