# Supplementary material for: The Role of Chromatid Interference in Determining Meiotic Crossover Patterns
Source: Front Plant Sci. 2021 Mar 9;12:656691. doi: 10.3389/fpls.2021.656691 (PMC7985435; doi:10.3389/fpls.2021.656691)
Supplement: Supplementary file 3 [file Table_3.DOCX]

**Supplementary Table S3. Analysis of chromatid interference (CI) in Arabidopsis male meiosis using sequencing-based genotyping data of 22 tetrads (Wijnker et al., 2013).** CI is determined using both the 2S:3S:4S DCO ratio method and the CI value. Results are shown for DCOs along the chromosomes, as well as for single-arm DCOs and for DCOs spanning a centromere. Deviations from the expected 1:2:1 ratio were statistically tested using a Chi-Square test of goodness-of-fit (when total number of DCOs ≥ 20) and via an exact multinomial test (when total number of DCOs < 20). Deviations of the CI value were statistically tested using the Wilcoxon signed rank test. Statistical tests were corrected via multiple penalty testing using Bonferroni correction (α = 0.008). Significant results before correcting are indicated with an asterisk.

|  | **WHOLE CHROMOSOME** | | | **SAME ARM** | | | | **DIFFERENT ARM** | | | |
| --- | --- | --- | --- | --- | --- | --- | --- | --- | --- | --- | --- |
|  | **Total**  **DCOs** | **Observed**  **2S:3S:4S ratio**  **(Expected ratio)** | **CI**  **value** | | **Total**  **DCOs** | **Observed**  **2S:3S:4S ratio**  **(Expected ratio)** | **CI**  **value** | | **Total**  **DCOs** | **Observed**  **2S:3S:4S ratio**  **(Expected ratio)** | **CI value** |
| **Chr1** | 21 | 3:11:7  (5.25:10.5:5.25) | 0.19 | | 8 | 0:4:4  (2:4:2) | 0.50 (*) | | 13 | 3:7:3  (3.25:6.5:3.25) | 0 |
|  |  | p-value  0.4556 | p-value  0.1136 | |  | p-value  0.1421 | p-value  0.03593 | |  | p-value  1 | p-value  1 |
| **Chr2** | 12 | 1:6:5  (3:6:3) | 0.33 | | 9 | 1:4:4  (2.25:4.5:2.25) | 0.33 | | 3 | 0:2:1  (0.75:1.5:0.75) | 0.33 |
|  |  | p-value  0.2816 | p-value  0.06472 | |  | p-value  0.4565 | p-value  0.1165 | |  | p-value  1 | p-value  0.5 |
| **Chr3** | 12 | 7:4:1 (*)  (3:6:3) | -0.50 (*) | | 1 | 1:0:0  (0.25:0.5:0.25) | -1 | | 11 | 6:4:1  (2.75:5.5:2.75) | -0.46 (*) |
|  |  | p-value  0.0467 | p-value  0.02054 | |  | p-value  0.5 | p-value  0.5 | |  | p-value  0.1166 | p-value  0.0336 |
| **Chr4** | 7 | 1:3:3  (1.75:3.5:1.75) | 0.29 | | 5 | 1:2:2  (1.25:2.5:1.25) | 0.2 | | 2 | 0:1:1  (0.5:1:0.5) | 0.50 |
|  |  | p-value  0.6104 | p-value  0.2119 | |  | p-value  0.8437 | p-value  0.3864 | |  | p-value  1 | p-value  0.5 |
| **Chr5** | 14 | 3:10:1  (3.5:7:3.5) | -0.14 | | 3 | 0:2:1  (0.75:1.5:0.75) | 0.33 | | 11 | 3:8:0  (2.75:5.5:2.75) | -0.27 |
|  |  | p-value  0.231 | p-value  0.2119 | |  | p-value  1 | p-value  0.5 | |  | p-value  0.1569 | p-value  0.07446 |
| **Total** | 66 | 15:34:17  (16.5:33:16.5) | 0.03 | | 26 | 3:12:11  (6.5:13:6.5) | 0.31 (*) | | 40 | 12:22:6  (10:20:10) | -0.15 |
|  |  | p-value  0.9131 | p-value  0.3659 | |  | p-value  0.07899 | p-value  0.01776 | |  | p-value  0.3329 | p-value  0.08236 |
