# Supplementary material for: The Role of Chromatid Interference in Determining Meiotic Crossover Patterns
Source: Front Plant Sci. 2021 Mar 9;12:656691. doi: 10.3389/fpls.2021.656691 (PMC7985435; doi:10.3389/fpls.2021.656691)
Supplement: Supplementary file 4 [file Table_4.DOCX]

**Supplementary Table S4. Analysis of chromatid interference (CI) in Arabidopsis male meiosis using sequencing-based genotyping data of 13 tetrads (Liu et al., 2018).** CI is determined using both the 2S:3S:4S DCO ratio method and the CI value. Results are shown for DCOs along the chromosomes, as well as for single-arm DCOs and for DCOs spanning a centromere. Deviations from the expected 1:2:1 ratio were statistically tested using a Chi-Square test of goodness-of-fit (when total number of DCOs ≥ 20) and via an exact multinomial test (when total number of DCOs < 20). Deviations of the CI value were statistically tested using the Wilcoxon signed rank test. Statistical tests were corrected via multiple penalty testing using Bonferroni correction (α = 0.008). Significant results before correcting are indicated with an asterisk.

|  | **WHOLE CHROMOSOME** | | | **SAME ARM** | | | **DIFFERENT ARM** | | |
| --- | --- | --- | --- | --- | --- | --- | --- | --- | --- |
|  | **Total**  **DCOs** | **Observed**  **2S:3S:4S ratio**  **(Expected ratio)** | **CI**  **value** | **Total**  **DCOs** | **Observed**  **2S:3S:4S ratio**  **(Expected ratio)** | **CI**  **value** | **Total**  **DCOs** | **Observed**  **2S:3S:4S ratio**  **(Expected ratio)** | **CI value** |
| **Chr1** | 18 | 6:6:6  (4.5:9:4.5) | 0 | 9 | 4:3:2  (2.25:4.5:2.25) | -0.22 | 9 | 2:3:4  (2.25:4.5:2.25) | 0.22 |
|  |  | p-value  0.2886 | p-value  1 |  | p-value  0.4564 | p-value  0.242 |  | p-value  0.4565 | p-value  0.242 |
| **Chr2** | 8 | 4:3:1  (2:4:2) | -0.38 | 1 | 0:0:1  (0.25:0.5:0.25) | 1 | 7 | 4:3:0  (1.75:3.5:1.75) | -0.57 (*) |
|  |  | p-value  0.4053 | p-value  0.117 |  | p-value  0.5 | p-value  0.5 |  | p-value  0.1147 | p-value  0.036 |
| **Chr3** | 9 | 2:3:4  (2.25:4.5:2.25) | 0.22 | 2 | 0:1:1  (0.5:1:0.5) | 0.50 | 7 | 2:2:3  (1.75:3.5:1.75) | 0.14 |
|  |  | p-value  0.4565 | p-value  0.242 |  | p-value  1 | p-value  0.5 |  | p-value  0.4736 | p-value  0.383 |
| **Chr4** | 5 | 2:3:0  (1.25:2.5:1.25) | -0.40 | 3 | 1:2:0  (0.75:1.5:0.75) | -0.33 | 2 | 1:1:0  (0.5:1:0.5) | -0.50 |
|  |  | p-value  0.6094 | p-value  0.173 |  | p-value  1 | p-value  0.5 |  | p-value  1 | p-value  0.5 |
| **Chr5** | 16 | 4:8:4  (4:8:4) | 0 | 7 | 0:5:2  (1.75:3.5:1.75) | 0.29 | 9 | 4:3:2  (2.25:4.5:2.25) | -0.22 |
|  |  | p-value  1 | p-value  1 |  | p-value  0.3711 | p-value  0.173 |  | p-value  0.4565 | p-value  0.242 |
| **Total** | 56 | 18:23:15  (14:28:14) | -0.05 | 22 | 5:11:6  (5.5:11:5.5) | 0.05 | 34 | 13:12:9  (8.5:17:8.5) | -0.12 |
|  |  | p-value  0.349 | p-value  0.304 |  | p-value  0.956 | p-value  0.401 |  | p-value  0.144 | p-value  0.202 |
