# Supplementary material for: The Role of Chromatid Interference in Determining Meiotic Crossover Patterns
Source: Front Plant Sci. 2021 Mar 9;12:656691. doi: 10.3389/fpls.2021.656691 (PMC7985435; doi:10.3389/fpls.2021.656691)
Supplement: Supplementary file 5 [file Table_5.DOCX]

**Supplementary Table S5. Analysis of chromatid interference (CI) in Arabidopsis male meiosis. using the merged dataset of three individual datasets (Copenhaver et al., 1998; Wijnker et al., 2013; Liu et al., 2018).** CI is determined using both the 2S:3S:4S DCO ratio method and the CI value. Results are shown for DCOs along the chromosomes, as well as for single-arm DCOs and for DCOs spanning a centromere. Deviations from the expected 1:2:1 ratio were statistically tested using a Chi-Square test of goodness-of-fit (when total number of DCOs ≥ 20) and via an exact multinomial test (when total number of DCOs < 20). Deviations of the CI value were statistically tested using the Wilcoxon signed rank test. Statistical tests were corrected via multiple penalty testing using Bonferroni correction (α = 0.008). Significant results before correcting are indicated with an asterisk.

|  | **WHOLE CHROMOSOME** | | | **SAME ARM** | | | **DIFFERENT ARM** | | |
| --- | --- | --- | --- | --- | --- | --- | --- | --- | --- |
|  | **Total**  **DCOs** | **Observed**  **2S:3S:4S ratio**  **(Expected ratio)** | **CI**  **value** | **Total**  **DCOs** | **Observed**  **2S:3S:4S ratio**  **(Expected ratio)** | **CI**  **value** | **Total**  **DCOs** | **Observed**  **2S:3S:4S ratio**  **(Expected ratio)** | **CI value** |
| **Chr1** | 140 | 42:61:37  (35:70:35) | -0.04 | 66 | 22:27:17  (16.5:33:16.5) | -0.08 | 74 | 20:34:20  (18.5:37:18.5) | 0 |
|  |  | p-value  0.263 | p-value  0.288 |  | p-value  0.230 | p-value  0.214 |  | p-value  0.794 | p-value  1 |
| **Chr2** | 118 | 29:61:28  (29.5:59:29.5) | -0.01 | 63 | 13:27:23  (15.75:31.5:15.75) | 0.16 (*) | 55 | 16:34:5 (*)  (13.75:27.5:13.75) | -0.20 (*) |
|  |  | p-value  0.927 | p-value  0.449 |  | p-value  0.108 | p-value  0.049 |  | p-value  0.024 | p-value  0.009 |
| **Chr3** | 87 | 29:40:18  (21.75:43.5:21.75) | -0.13 | 26 | 10:10:6  (6.5:13:6.5) | -0.15 | 61 | 19:30:12  (15.25:30.5:15.25) | -0.12 |
|  |  | p-value  0.188 | p-value  0.055 |  | p-value  0.270 | p-value  0.166 |  | p-value  0.444 | p-value  0.106 |
| **Chr4** | 81 | 23:34:24  (20.25:40.5:20.25) | 0.01 | 38 | 14:13:11  (9.5:10:9.5) | -0.08 | 43 | 9:21:13  (10.75:21.5:10.75) | 0.09 |
|  |  | p-value  0.348 | p-value  0.444 |  | p-value  0.119 | p-value  0.279 |  | p-value  0.681 | p-value  0.202 |
| **Chr5** | 81 | 22:39:20  (20.25:40.5:20.25) | -0.03 | 24 | 4:12:8  (6:12:6) | 0.17 | 57 | 18:27:12  (14.25:28.5:14.25) | -0.11 |
|  |  | p-value  0.9 | p-value  0.382 |  | p-value  0.513 | p-value  0.133 |  | p-value  0.491 | p-value  0.139 |
| **Total** | 507 | 145:235:127  (126.75:253.5:126.75) | -0.04 | 217 | 63:89:65 (*)  (54.25:108.5:54.25) | 0.01 | 290 | 82:146:62  (72.5:145:72.5) | -0.07 (*) |
|  |  | p-value  0.137 | p-value  0.138 |  | p-value  0.030 | p-value  0.430 |  | p-value  0.250 | p-value  0.048 |
