# Supplementary material for: The Role of Chromatid Interference in Determining Meiotic Crossover Patterns
Source: Front Plant Sci. 2021 Mar 9;12:656691. doi: 10.3389/fpls.2021.656691 (PMC7985435; doi:10.3389/fpls.2021.656691)
Supplement: Supplementary file 6 [file Table_6.DOCX]

**Supplementary Table S6. P-values for analysis of chromatid interference (CI) in maize male meiosis.** P-values are shown for both the 2S:3S:4S DCO ratio (Chi-Square test of goodness-of-fit when total number of DCOs ≥ 20 and exact multinomial test when total number of DCOs < 20) and the CI value (Wilcoxon signed rank test), for all DCOs along the chromosomes, as well as for single-arm DCOs and for DCOs spanning a centromere. P-values correspond to data represented in Table 2. Results are based on data from Li et al. (2015).

|  | **WHOLE CHROMOSOME** | | **SAME ARM** | | **DIFFERENT ARM** | |
| --- | --- | --- | --- | --- | --- | --- |
|  | **p-value**  **2S:3S:4S ratio** | **p-value**  **CI value** | **p-value**  **2S:3S:4S ratio** | **p-value**  **CI value** | **p-value**  **2S:3S:4S ratio** | **p-value**  **CI value** |
| **Chr1** | 0.5956 | 0.1694 | 0.6208 | 0.3183 | 0.3679 | 0.1507 |
| **Chr2** | 0.4597 | 0.3728 | 0.3132 | 0.06696 | 0.05882 | 0.02054 |
| **Chr3** | 0.01069 | 0.2207 | 0.04623 | 0.3276 | 0.05882 | 0.02054 |
| **Chr4** | 0.6694 | 0.3659 | 0.1271 | 0.05671 | 0.2773 | 0.08897 |
| **Chr5** | 0.4055 | 0.3043 | 0.0747 | 0.1305 | 0.6065 | 0.3089 |
| **Chr6** | 0.1006 | 0.1329 | 0.0484 | 0.03945 | 0.3227 | 0.1493 |
| **Chr7** | 0.6175 | 0.2989 | 0.4301 | 0.2529 | 1 | 1 |
| **Chr8** | 0.4442 | 0.1714 | 0.8855 | 0.3276 | 0.3247 | 0.1753 |
| **Chr9** | 0.9006 | 0.3417 | 0.8607 | 0.3949 | 0.7873 | 0.406 |
| **Chr10** | 0.487 | 0.1305 | 0.749 | 0.2827 | 0.3927 | 0.1753 |
| **Total** | 0.1845 | 0.4555 | 0.1871 | 0.3152 | 0.5572 | 0.1926 |
