# Supplementary material for: The Role of Chromatid Interference in Determining Meiotic Crossover Patterns
Source: Front Plant Sci. 2021 Mar 9;12:656691. doi: 10.3389/fpls.2021.656691 (PMC7985435; doi:10.3389/fpls.2021.656691)
Supplement: Supplementary file 8 [file Image_1.PDF]

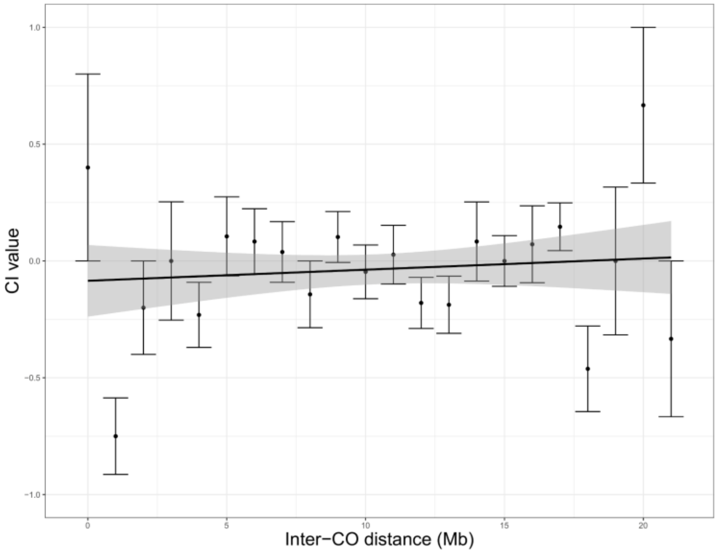

Supplementary Figure S1. Chromatid interference (CI) in function of physical distance (Mb) between adjacent COs (inter-CO distance) in Arabidopsis male meiosis using both sequencing- and PCR-based genotyping data. Linear regression is performed with the total number of DCOs per inter-CO distance as weighted factors. Grey shaded areas indicate the 95% confidence interval for the regression line. Intercept = -0.0849; Slope = 0.00476; R^2 = 0.001. Results are based on the merged dataset from PCR-based (Copenhaver et al., 1998) and sequencing-based (Wijnker et al., 2013; Liu et al., 2018) tetrad genotyping data.
